# Seed Dormancy Class and Germination Characteristics of *Prunus spachiana* (Lavallée ex Ed.Otto) Kitam. f. *ascendens* (Makino) Kitam Native to the Korean Peninsula

**DOI:** 10.3390/plants13040502

**Published:** 2024-02-11

**Authors:** Gun Mo Kim, Chung Ho Ko, Jae Min Chung, Hak Cheol Kwon, Yong Ha Rhie, Seung Youn Lee

**Affiliations:** 1 Department of Horticulture and Breeding, Graduate of Andong National University, Andong 36792, Republic of Korea; gsh03185@naver.com; 2 Division of Wild Plant Seed Research, Baekdudaegan National Arboretum, Bonghwa 36209, Republic of Korea; 3 Garden and Plant Resources Division, Korea National Arboretum, Yangpyeong 12519, Republic of Korea; tune0820@korea.kr (C.H.K.); rhuso@korea.kr (J.M.C.); 4 Natural Product Informatics Research Center, Korea Institute of Science and Technology, Gangneung 25451, Republic of Korea; hkwon@kist.re.kr; 5 Department of Horticulture and Forestry, Pai Chai University, Daejeon 35345, Republic of Korea; rhie@pcu.ac.kr; 6 Department of Smart Horticultural Science, Andong National University, Andong 36792, Republic of Korea

**Keywords:** endocarp, gibberellin, H_2_SO_4_, phenology, *Prunus*, seed dormancy

## Abstract

*Prunus spachiana* (Lavallée ex Ed.Otto) Kitam. f. *ascendens* (Makino) Kitam leaves exert natural anti-inflammatory effects by inhibiting nitric oxide formation. *P. spachiana* flowers bloom earlier than other *Prunus* spp. and thus could serve as a valuable resource for the horticulture and pharmaceutical industries. However, its seed dormancy class and germination traits remain uncharacterized. Thus, this study aimed to characterize the seed dormancy and germination of *P. spachiana*. Imbibition, phenological, and move-along experiments were performed, and the effects of H_2_SO_4_ treatment, hormone soaking, warm/cold stratification, and endocarp removal on germination were explored. Observation revealed that ripe seeds of *P. spachiana* contain developed embryos and are water permeable. Radicle and shoot emergence began in March and April, respectively, under natural conditions in the year following production. No seed germination was observed after 30 days of incubation at 4, 15/6, 20/10, or 25/15 °C under light/dark conditions, indicating the physiological dormancy of the seeds. Germination increased with prolonged stratification and was affected by incubation temperature. Seed scarification by H_2_SO_4_ and soaking with gibberellic acid (GA_3_) and fluridone were ineffective in breaking dormancy. However, GA_3_ soaking of the seeds after endocarp removal effectively induced germination (100%). These results indicate that *P. spachiana* seeds exhibit intermediate physiological dormancy.

## 1. Introduction

Seed dormancy is one of the survival strategies for plants to survive in nature. Seed dormancy can be classified as primary or secondary, based on when dormancy is induced [1]. Primary dormancy is induced during seed development by endogenous factors and/or environmental conditions experienced by the mother plant [2]. Primary dormancy is further categorized into five types: physiological (PD), morphological (MD), morphophysiological (MPD), physical (PY), and combinational [3,4]. Among these types, PD is the most common class of seed dormancy on Earth [3]. PD can be broken by warm and/or cold stratification, or after-ripening [5].

PD has three levels: nondeep, intermediate, and deep. Depending on the species, nondeep PD is broken by warm or cold stratification, and the seeds of many species can break dormancy slowly during dry storage at room temperature (after-ripening) [6]. In temperate regions, cold stratification for 3–4 and 2–3 months can break deep and intermediate PD, respectively, but a period of warm stratification or after-ripening can shorten the cold stratification period required to break intermediate but not deep PD. In addition, gibberellic acid (GA_3_) can promote the germination of seeds with nondeep and intermediate PD but not that of seeds with deep PD [6,7]. Therefore, if we classify the type of dormancy that a certain plant’s seeds have, we can effectively break down seed dormancy and promote germination, which can be used in practical propagation program.

*Prunus* species are widely cultivated in different parts of the world [8]. In Korea, 22 *Prunus* species are distributed, including the cultivated ones [9]. *P. spachiana* flowers bloom before the leaves in April and earlier than other *Prunus* species, indicating an important breeding source. Their morphological characteristics are 1.5–1.8 cm in diameter, pale pink, 2–5 flowers hanging in umbel, and 8–10 mm long pedicels, with hair. The fruit is round and ripens to black in June–July. Its native region is the southern part of the Korean Peninsula [10]. *P. spachiana* is a deciduous broad-leaved arboreous tree with a height of approximately 10 m and leaves that are long ovate, 6–12 cm long, 3–4 cm wide, membranous, and have double saw teeth [11]. *Prunus* has been used in folk medicine since ancient times because of its anti-inflammatory, antihyperlipidemic, and anticancer activities [12,13]. Anti-inflammatory substances are present in *P. spachiana* leaves [14,15]. Consequently, *P. spachiana* leaves could be used as plant-derived agents in the pharmaceutical and cosmetic industries. *P. spachiana* is the maternal line of *Prunus* × *nudiflora*, a plant endemic to Jeju Island, an island located in the southwest of the Korean Peninsula [16]. *P. spachiana* is generally propagated by grafting. However, the grafting rate is very low [17]. Seed propagation has various advantages. In a plant breeding program, hybrids are first raised from seeds; thus, seeds are the most important means of developing new cultivars [18]. Seeds also offer a convenient method for the long-term storage and convenient transportation of plants. When stored properly, seeds may be viable for very long periods [18]. Therefore, seed propagation is advantageous. However, many wild plants have seed dormancy that prevents them from germinating for a certain period of time even under appropriate environmental conditions, making seed propagation difficult [3,4].

*P. prunus* seeds require prolonged cold stratification to break dormancy [19]. *Prunus campanulata* seeds stratified at 4 °C for 12 weeks germinate to 88% [20]. In this experiment, it was reported that seed dormancy was effectively broken by treatment with fluridone, an inhibitor of abscisic acid (ABA) biosynthesis. Therefore, it can be assumed that ABA may play an important role in seed dormancy of other species of the genus *Prunus*. *Prunus mabaleb* L. seeds germinated to 33% after only 4 months of cold stratification [21]. *Prunus yedoensis* seeds that are placed in moistened sand at 4 °C for either 3 or 6 months did not germinate [22]. These results suggest that the cold stratification period for breaking dormancy differs among *Prunus* species. However, the seed dormancy and germination of *P. spachiana* seeds are yet to be characterized. Therefore, in order to more clearly understand the seed dormancy of plants of the genus *Prunus*, various treatments related to ABA biosynthesis as well as high- and low-temperature treatments should be applied.

Thus, the present study aimed to determine the dormancy and germination requirements of *P. spachiana*. Specifically, we investigated the following: (1) temperature requirements for dormancy breaking, (2) phenology related to germination and seedling emergence, (3) the effects of H_2_SO_4_ and GA_3_ on radicle emergence, and (4) the effects of endocarp removal on germination.

## 2. Results

### 2.1. Seed Traits

**Figure 1:**
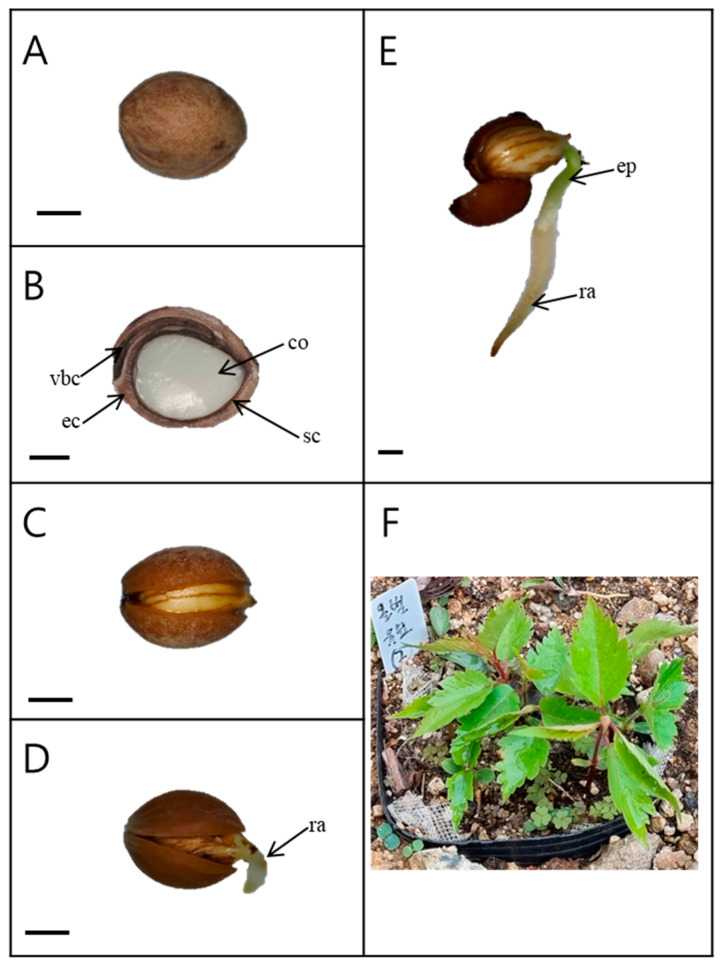
Germination and seedling development of *Prunus spachiana*. Bars = 2 mm. (**A**) Seed external morphology; (**B**) longitudinal section of a seed; (**C**) an opened endocarp; (**D**) radicle emergence; (**E**) a radicle and an epicotyl; and (**F**) seedlings. ec, endocarp; sc, seed coat; co, cotyledons; vbs, vascular bundle canal; ra, radicle; and ep, epicotyl. In Figure 1F, the white label indicates a replication for investigation of seedling emergence.

The seeds (with endocarp) were light pink in color and spherical (Figure 1A). The length and width of the seeds harvested in 2019 were 7.11 ± 0.09 and 5.52 ± 0.07 mm, respectively. The weight of 100 seeds was 8.87 ± 0.04 g (Table 1). The interior of the seed consisted of a large, fully developed embryo (Figure 1B). As the endocarp of the seed opened, the root emerged (Figure 1D).

### 2.2. Imbibition and Staining Tests

**Figure 2:**
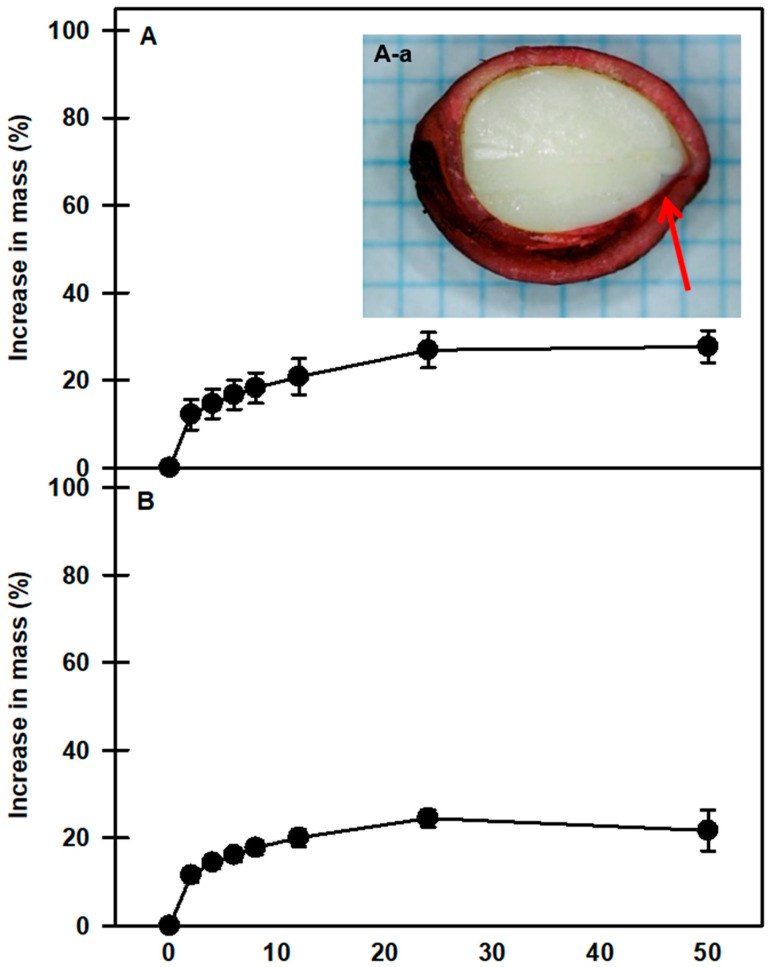
Water uptake by nonscarified seeds (**A**) and scarified seeds (**B**) of *Prunus spachiana* as represented by an increase in mass. Seeds were incubated at room temperature (20–25 °C) on filter paper with distilled water for 50 h. Seeds were soaked in Safranin O (1%) for 24 h and absorbed through the vascular bundle canal (**A-a**). The arrow shows that the dye has moved to the bottom of the seed interior. Vertical bars indicate the standard error of the means (n = 3).

At room temperature, mechanically scarified and nonscarified seeds of *P. spachiana* showed similar imbibition patterns. After 24 h, the weights of the nonscarified and scarified seeds increased by 24.4 ± 1.91% and 26.9 ± 3.9%, respectively, compared with their initial masses (Figure 2). The vascular bundle canal area inside the seed was stained with Safranin O (Figure 2(A-a)), which means that moisture can move into the seed through the dyed area.

### 2.3. Phenological Experiment

**Figure 3:**
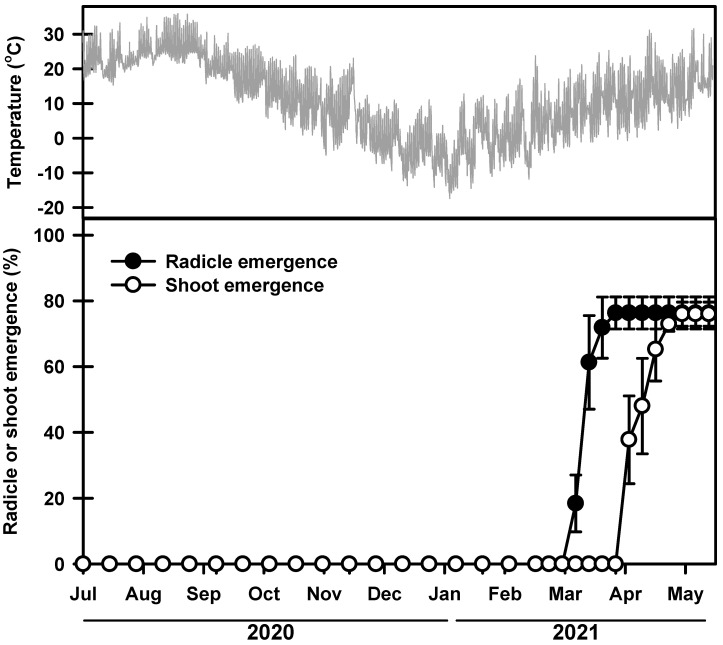
Mean daily soil temperatures and phenology of germination and shoot emergence from seeds sown on field soil. Error bars indicate the mean ± SE of three replications.

The germination rate started to increase on 11 and 25 March 2021, and during this time, the average daily maximum and minimum soil temperatures were 18.7 and 2.3 °C, respectively (Figure 3). The seeds had a final germination rate of 76.3% on 20 May 2021. Most of the seedlings emerged between 8 and 23 April 2021. Then, 75.9% of the seedlings emerged on 20 May 2021. No additional seedlings emerged thereafter.

### 2.4. Move-Along Experiment

**Figure 4:**
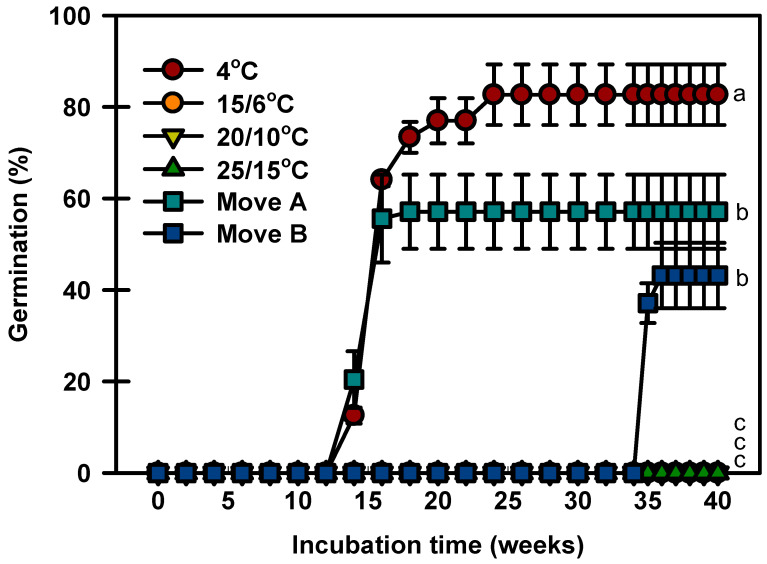
Germination of *Prunus spachiana* seeds incubated under two temperature sequences and four constant temperatures. Move A: 4 °C (12 weeks) → 15/6 °C (4 weeks) → 20/10 °C (4 weeks) → 25/15 °C (20 weeks). Move B: 25/15 °C (12 weeks) → 20/10 °C (4 weeks) → 15/6 °C (4 weeks) → 4 °C (14 weeks) → 15/6 °C (4 weeks) → 20/10 °C (2 weeks). Error bars indicate the mean ± SE of three replications. Means with significant differences were compared using Duncan’s multiple-range tests at *p* ≤ 0.05.

Seeds germinated to 82.6% at 4 °C after constant incubation, whereas no seeds germinated at 15/6, 20/10, or 25/15 °C at 40 weeks (Figure 4). In Move A, the seeds started to germinate after 14 weeks of incubation and quickly increased to 57.1% during incubation. In Move B, no seeds germinated until they were transferred to 4 °C. After being transferred to 15/6 °C, the germination of seeds reached 43.1% and did not increase further.

### 2.5. H_2_SO_4_ and GA_3_ Experiment

**Figure 5:**
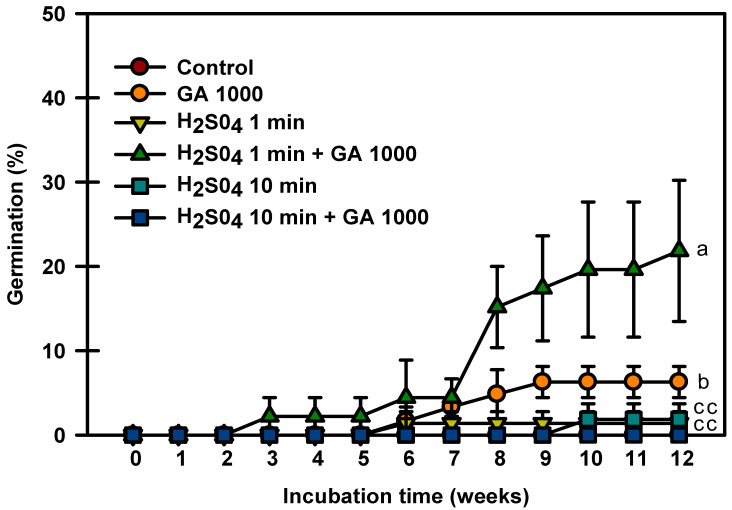
Germination of *Prunus spachiana* seeds treated with H_2_SO_4_ (0, 1, or 10 min) and GA_3_ (1000 mg·L^−1^). Error bars indicate the mean ± SE of three replications. Means with significant differences were compared using Duncan’s multiple-range tests at *p* ≤ 0.05.

Control seeds did not germinate in this experiment, and the seeds treated with only H_2_SO_4_ for 1 and 10 min germinated to 1.3 and 1.8%, respectively (Figure 5). The seeds treated with H_2_SO_4_ 1 min + GA_3_ 1000 mg⋅L^−1^ had the highest germination rate of 21.8%. The seeds treated with H_2_SO_4_ 10 min + GA_3_ 1000 mg⋅L^−1^ did not germinate. The above experiments showed that treatment with H_2_SO_4_ + GA_3_ influenced seed germination.

### 2.6. GA_3_ and Fluridone Experiment

**Figure 6:**
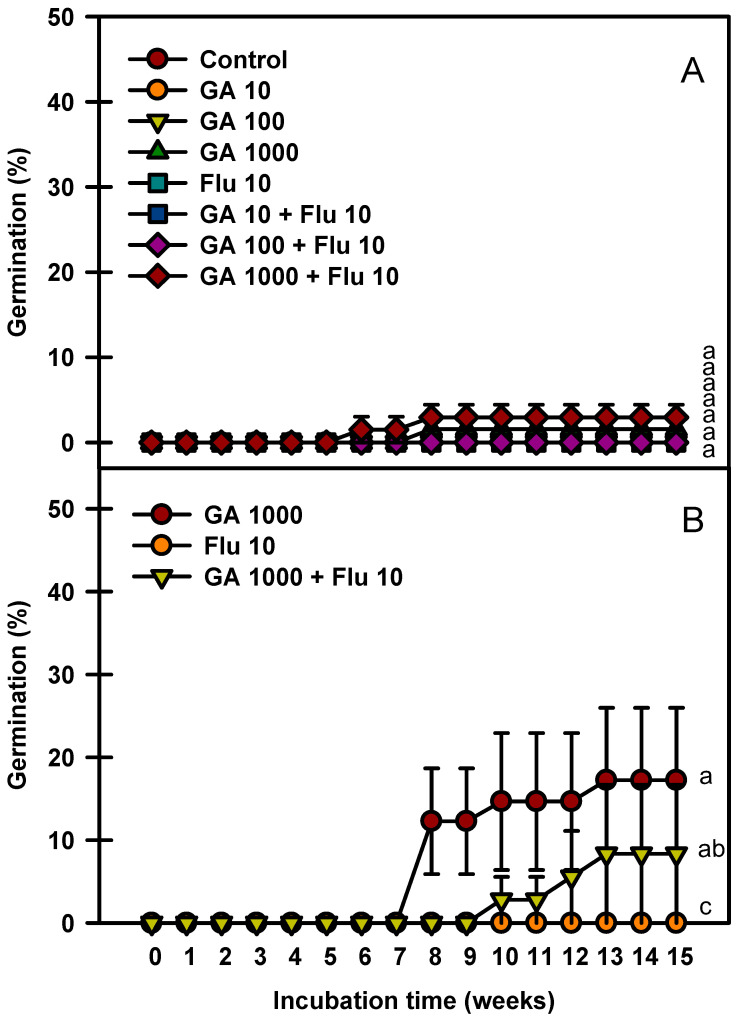
Germination of *Prunus spachiana* seeds treated with GA_3_ (0, 10, 100, and 1000 mg·L^−1^) and fluridone (10 mg·L^−1^). GA_3_ 24 h soaking (**A**) and GA_3_ 48 h soaking (**B**). Error bars indicate the mean ± SE of three replications. Means with significant differences were compared using Duncan’s multiple-range tests at *p* ≤ 0.05.

The seed germination rate increased as the immersion time of GA_3_ was prolonged (Figure 6). Fluridone treatment alone at 10 mg ⋅ L^−1^ did not affect the seed germination rate. The seeds immersed in GA_3_ 1000 mg⋅L^−1^ + fluridone 10 mg⋅L^−1^ for 24 h germinated to 2.9% (Figure 6A), whereas those immersed in GA_3_ 1000 mg·L^−1^ for 48 h germinated to 17.9% (Figure 6B).

### 2.7. Warm and/or Cold Stratification Experiment

**Figure 7:**
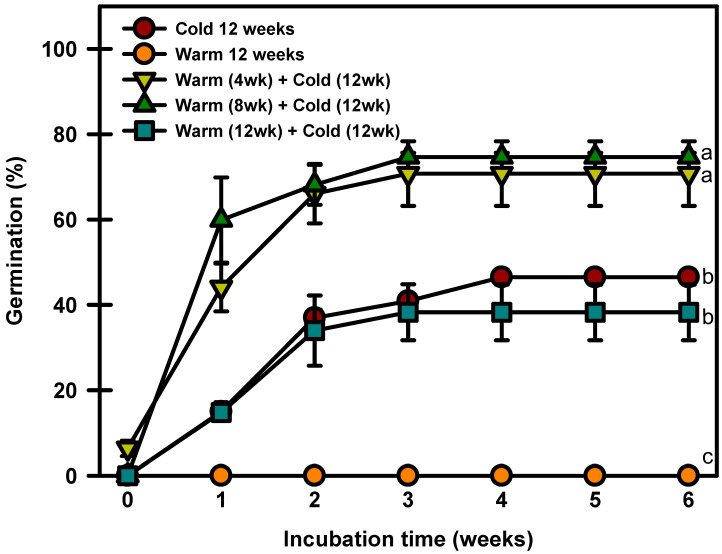
Germination of *Prunus spachiana* seeds treated with cold (4 °C), warm (25/15 °C), and warm + cold stratification. The seeds were incubated at 20/10 °C after stratification. Error bars indicate the mean ± SE of three replications. Means with significant differences were compared using Duncan’s multiple-range tests at *p* ≤ 0.05.

The seeds treated with warm stratification only did not break dormancy (Figure 7). The seeds cold stratified for 12 weeks germinated to 46.4% at 20/10 °C. The seeds warm stratified at 25/15 °C for 4, 8, or 12 weeks and then cold stratified at 4 °C germinated to 70.7, 74.6, or 38.2%, respectively, after 6 weeks of incubation at 20/10 °C.

### 2.8. Removal of Endocarp

**Figure 8:**
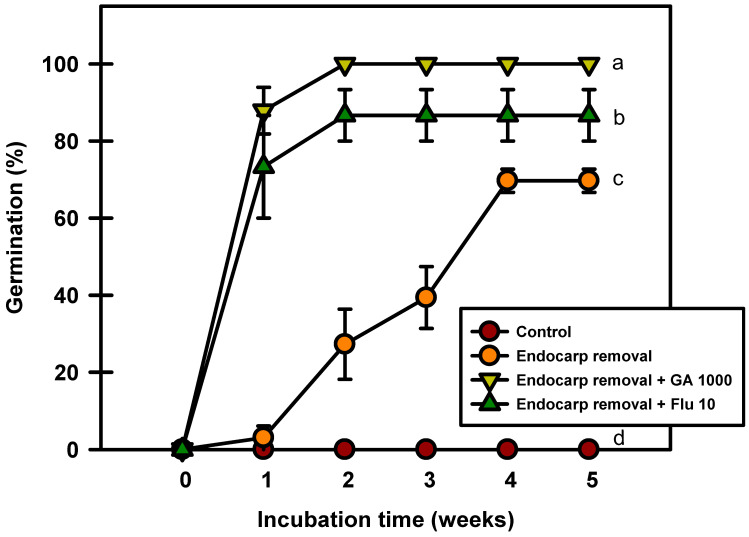
Germination of *Prunus spachiana* seeds after endocarp removal and 1000 mg·L^−1^ GA_3_ or 10 mg·L^−1^ fluridone soaking. Error bars indicate the mean ± SE of three replications. Means with significant differences were compared using Duncan’s multiple-range tests at *p* ≤ 0.05.

Seed germination occurred after the endocarp was removed (Figure 8). The seeds with removed endocarps germinated to 69.6% after 5 weeks of incubation at 20/10 °C. However, the seeds treated with GA_3_ and fluridone after endocarp removal germinated to 100% and 86.6%, respectively. The results of this experiment confirmed that the germination rate increased when the endocarp was removed before incubation.

## 3. Discussion

To understand how *P. spachiana* adapts to its natural habitat, we investigated its seed dormancy and germination under different conditions. Fresh *P. spachiana* seeds buried in the summer of 2020 germinated next spring (Figure 3); none of the seeds germinated at 15/6, 20/10, and 25/15 °C under laboratory conditions (Figure 4), indicating that the *P. spachiana* seeds remained dormant when freshly dispersed. Therefore, *P. spachiana* seeds exhibit PD.

Physically dormant seeds fail to imbibe water when placed on wet substrates [5,6]. In the present study, nonscarified or scarified *P. spachiana* seeds were imbibed with a small amount of water (≥24%). However, safranin O staining results confirmed that moisture was absorbed through the vascular bundle canal, which indicated that the seeds were permeable to water (Figure 2). Furthermore, the seeds did not have an underdeveloped embryo that had to grow prior to radicle emergence. PY is caused by one or more water-impermeable layers in the seed or fruit coat. In seeds with (PY + PD), the seed (or fruit) coat is water impermeable, and, in addition, the embryo is physiologically dormant at harvest [3,4]. Thus, the results of this study indicate that they did not exhibit physical or morphological dormancy.

Acid treatments are often used to break down thick hard seed coats [23]. The scarification of *Rhynchosia capitata* seeds with H_2_SO_4_ induces seed germination. The seed germination percentage increases with the prolonged soaking time (up to 80 min) but decreases with the further prolonged soaking time (>80 min) [24]. For *Prunus mahaleb*, the germination percentage of nonstratified seeds that had not been subjected to scarification with H_2_SO_4_ is low [25]. Treatment with H_2_SO_4_ alone was ineffective in breaking the dormancy of *P. spachiana* seeds. However, the germination rate was higher when treated with GA_3_ after scarification treatment than when treated with GA_3_ alone (Figure 5). This means that the endocarp affects seed dormancy.

ABA plays a pivotal role in the development of primary dormancy, and gibberellins (GAs) are involved in the induction of germination [26]. In PD, seed germination is regulated by quantitative changes in and sensitivity to GAs and ABA [27]. *P. yedoensis* seeds treated with GA_3_ germinate between 29 and 63% [22]. In the present study, *P. spachiana* seeds were little affected by immersion in GA_3_ for 24 h, but germination was promoted when treated for 48 h (Figure 6). Similarly, the germination rate of *P. campanulate* seeds increases with prolonged GA_3_ immersion [20]. However, GA_3_ was not very effective in enhancing germination even when vacuum infiltration was used to facilitate hormone uptake. The physical constraint imposed by the thick endocarp (1.03 mm thickness) possibly blocked the entrance of GAs into the seeds. Furthermore, the high concentration of ABA in the endocarp may inhibit radicle protrusion [20]. In the present study, the germination rate of *P. spachiana* seeds increased with prolonged GA_3_ treatment, but this effect was further enhanced when the endocarp was removed. This result proved that the endocarp can inhibit germination.

Cold stratification has been widely used as a presowing treatment to break seed dormancy and optimize the germination of dormant seeds in many species [28]. *P. spachiana* seeds germinated even after 12 weeks of cold stratification (Figure 7). According to previous studies on the genus *Prunus*, cold moist stratification alone also stimulated a high percentage of seeds to germinate [20]. However, *P. avium* L seeds after cold stratification for four months resulted in low germination rates [29]. Thus, even the same genus has different depths of dormancy. Nikolaeva [7] classified PD into nondeep, intermediate, and deep. Warm or cold stratification can break nondeep PD depending on the species, and GA_3_ is effective in promoting seed germination [27,30]. Intermediate PD is broken by long (2–3 months) periods of cold stratification [3,4]. In addition, performing warm stratification before cold stratification can promote seed germination [31]. In addition, GA_3_ promotes germination in some (but not all) species on the intermediate PD [3,4]. Deep PD is broken by long (3–4 months) periods of cold stratification, and GA_3_ is not effective in promoting seed germination [3,4]. In the present study, *P. spachiana* required approximately 3 months of cold stratification to break dormancy, and GA_3_ influenced seed germination. In addition, warm stratification followed by cold stratification further increased the seed germination rate (Figure 7). These results suggest that *P. spachiana* seeds exhibit intermediate PD.

The seed coat provides a physical impediment, commonly referred to as mechanical resistance, to germination [32]. In the present study, endocarp removal alone resulted in a germination rate of 70%. However, endocarp removal followed by treatment with GA_3_ or fluridone further increased this rate to over 80% within 2 weeks (Figure 8). In *P. campanulata* seeds, endocarp removal gradually increases germination percentage, with only 25% of the seeds germinated within 21 days. Seeds with endocarp removed and treated with 0.26 mM GA_3_ germinate to 90% after 21 days [20]. The order of ABA concentration in fresh matured seeds was endocarp > seed coat > embryo, and its concentration in endocarp plus seed coat was about 6.2-fold higher than that in embryo [20]. In *P. spachiana* seeds, germination inhibition by the endocarp was approximately 70%, and that by true seeds was 30%. This experiment suggests that endocarps in seeds can inhibit germination. Therefore, it can be assumed that ABA in the covering layers had a significant effect on seed dormancy and germination in *P. spachiana*. However, in this study, fluridone treatment at a concentration of 10 mg·L^−1^ in the presence of endocarp was not effective in promoting germination (Figure 6). The balance between ABA and GA is very important in inducing the end of seed dormancy and the start of germination [27], and the effect of fluridone treatment concentration to inhibit ABA biosynthesis varies depending on the plant species [20,33]. In this study, only a single concentration of fluridone was used, so experiments with various concentrations of fluridone in the presence of endocarp need to be conducted.

## 4. Materials and Methods

### 4.1. Seed Collection

The seeds of *P. spachiana* were collected from the Korea National Arboretum on 20 June 2019, and 4 June 2020. After harvesting, the seeds were dried in a laboratory at 20–23 °C for 2 weeks and then stored in a 0 °C refrigerator (DOI1815DOP; Winiamando, Gwangju, Korea). The seeds collected in 2019 were used for the move-along test, H_2_SO_4_ treatment, hormone treatment, warm or cold stratification, and endocarp removal experiments. Seeds collected in 2020 were used for phenological experiments.

### 4.2. Imbibition and Staining Tests

The permeability of the seed coat was determined by measuring the water absorption of mechanically scarified and nonscarified seeds under laboratory conditions (20–25 °C). Three replicates of 10 mechanically scarified (using a razor blade) or nonscarified seeds were used. The seeds were placed in 9 cm-diameter Petri dishes with two layers of filter paper moistened with distilled water. The fresh weight of the seeds was measured after 0, 3, 6, 9, 12, 24, and 50 h of incubation. Percent water uptake was calculated as % W_s_ = [(W_i_ − W_d_)/W_d_] × 100, where W_s_ is the increase in the mass of seed, W_i_ is the seed mass after a given interval of imbibition, and W_d_ is the initial seed mass at 0 h. For the observation of cross sections, the seeds were immersed in 1% safranin-O for 24 h. The seeds were observed using a USB microscope (AM3111 Dino-Lite Premier, ANMO Electronics Co., New Taipei City, Taiwan).

### 4.3. Phenological Experiment

This experiment was performed to monitor radicle and shoot emergence from seeds exposed to seasonal temperature changes under natural conditions. Three replicates of 20 seeds were buried in a shaded area in the experimental garden of Andong National University, Korea (36°32′40.41888″ N, 128°48′2.67516″ E), on 2 July 2020. The soil temperature at a depth of 3 cm was monitored using a temperature data logger (3683WD1, Spectrum Technologies, Inc., Plainfield, IL, USA) every hour during the experiment. For radicle emergence, the seeds were wrapped in a plastic mesh filled with sand and buried to a depth of 3 cm in a plastic pot filled with horticultural substrate (Sunshine Mix #4; SunGro Horticulture, Agawam, MA, USA). This plastic pot was buried at the ground level. Three replicates were prepared, each with 20 seeds per pot. Once the emerged radicle reached a length of 1 mm, the seeds were regarded as germinated and immediately removed. For shoot emergence, the seeds were buried to a depth of 3 cm in a plastic pot filled with horticultural substrate. This plastic pot was buried at the ground level. Three replicates were prepared, each with 20 seeds per pot. The experiment lasted for 10 months, during which the emerged seedlings were counted and removed each week.

### 4.4. Move-Along Experiment

This experiment simulated seasonal temperature changes with temperature sequences in incubators, which enabled us to verify whether warm and/or cold temperatures are required for radicle emergence from seeds. Three replicates of 20 fresh seeds were used in this study. On 13 July 2019, six treatments were started, with seeds being moved (→) in the following sequence: constant temperature at 4, 15/6, 20/10, and 25/15 °C for 40 weeks; Move A: 4 °C for 12 weeks → 15/6 °C for 4 weeks → 20/10 °C for 4 weeks → 25/15 °C for 20 weeks; and Move B: 25/15 °C for 12 weeks → 20/10 °C for 4 weeks → 15/6 °C for 4 weeks → 4 °C for 14 weeks → 15/6 °C for 4 weeks → 20/10 °C for 2 weeks. Three replicates of 20 seeds were placed in 9 cm-diameter Petri dishes on two layers of Whatman No. 1 filter paper. Parafilm was used to seal the Petri dishes to prevent water loss during incubation. Germination was tested through seed incubation with alternating 12 h photoperiods under light/dark conditions.

### 4.5. H_2_SO_4_ and GA_3_ Experiment

To determine the effects of GA_3_ treatment and various soaking times in H_2_SO_4_ on breaking seed dormancy, three replicates of 20 fresh seeds were soaked in H_2_SO_4_ for 0, 1, or 10 min. The seeds were washed with distilled water, soaked in 5 mL of 0 or 1000 mg·L^−1^ GA_3_ solution for 24 h, and then incubated at 20/10 °C under light/dark (12 h/12 h of light and dark, respectively). The germination percentage was calculated after 12 weeks of incubation.

### 4.6. GA_3_ and Fluridone Experiment

This experiment was conducted to determine the effects of different soaking times in GA_3_ and fluridone on breaking seed dormancy. Three replicates of 20 fresh seeds were soaked in 5 mL of 0, 10, 100, or 1000 mg·L^−1^ GA_3_ solution, 5 mL of 10 mg·L^−1^ fluridone solution, and the combination solution of GA_3_ and fluridone for 24 h. In addition, three replicates of 10 fresh seeds were soaked in 5 mL of 1000 mg·L^−1^ GA_3_ solution, 5 mL of 10 mg·L^−1^ fluridone solution, and their combination for 48 h. The treated seeds were incubated at 20/10 °C for 15 weeks, and the final germination rate was determined.

### 4.7. Warm and/or Cold Stratification Experiment

To determine the effects of warm and cold stratification on the breaking of seed dormancy, three replicates of 20 fresh seeds were used in this study. Seeds were subjected to warm and cold stratification for 12 weeks, warm stratification for 4 weeks + cold stratification for 12 weeks, warm stratification for 8 weeks + cold stratification for 12 weeks, and warm stratification for 12 weeks + cold stratification for 12 weeks. After treatment, incubation was performed at 20/10 °C for 6 weeks, and the final germination rate was determined.

### 4.8. Removal of Endocarp

This experiment was conducted to evaluate the effect of endocarp removal on seed dormancy. Three replicates of 10 fresh seeds were used. The seeds were used as controls, and the endocarp of the seeds was removed. True seeds (without endocarp) were soaked for 24 h in distilled water, GA_3_ 1000 mg·L^−1^, and fluridone 10 mg·L^−1^. After treatment, incubation was performed at 20/10 °C for five weeks, and the final germination rate was determined.

### 4.9. Statistical Analysis

The data were analyzed using Statistical Analysis System (SAS) version 9.4 (SAS Institute Inc., Cary, NC, USA). Means with significant differences were compared using Duncan’s multiple-range tests at *p* ≤ 0.05. Graphs were plotted using SigmaPlot 10.0 (SPSS Inc., Chicago, IL, USA).

## 5. Conclusions

*P. spachiana* seeds had intact, water-permeable seed coats and fully developed embryos, indicating that they did not undergo physical nor morphological dormancy. The seeds did not germinate within a month. The seeds can germinate after cold stratification for approximately 3 months, and GA_3_ treatment further promoted their germination. In addition, warm stratification followed by cold stratification further increased the seed germination rate and reduced the number of days required for germination. Therefore, they could be classified as PD, specifically intermediate PD. A high germination rate can be obtained in a short period by removing the endocarp and applying GA_3_ treatment. Germination inhibition by the endocarp was approximately 70%, and that by true seeds was about 30% in *P. spachiana* seeds. The results of this study will be of great help in fields that require mass propagation of seedlings. In addition, continued research is needed to classify dormancy types of yet unknown *Prunus* species.

## Figures and Tables

**Table 1:** Seed characteristics of *Prunus spachiana* (Lavallée ex Ed.Otto) Kitam. f. *ascendens* (Makino) Kitam in this study.

Scientific Name	Common Name	Collection Location	Collection Date	Length (mm)	Width (mm)	100 Seed Weight (g)
*Prunus spachiana* (Lavallée ex Ed.Otto) Kitam. f. *ascendens* (Makino) Kitam	Wild-spring cherry	Living collection from Korea National Arboretum	29 June 2019	7.11 ± 0.096 ^z^	5.52 ± 0.073	8.87 ± 0.049 ^y^
			8 June 2020	- ^x^	-	-

^z^ Mean ± standard error (n = 20). ^y^ Mean ± standard error (n = 5). ^x^ No data.

## Data Availability

Data are contained within the article.
